# Functional roles of hnRNPA2/B1 regulated by METTL3 in mammalian embryonic development

**DOI:** 10.1038/s41598-019-44714-1

**Published:** 2019-06-14

**Authors:** Jeongwoo Kwon, Yu-Jin Jo, Suk Namgoong, Nam-Hyung Kim

**Affiliations:** 1 Department of Animal Sciences, Chungbuk National University, Gaesin-dong, Cheongju, Chungbuk 361-763 Republic of Korea; 2 Primate Resources Center (PRC), Korea Research Institute of Bioscience and Biotechnology (KRIBB), Jeongeup-si, Jeollabuk-do 56216 Republic of Korea

**Keywords:** Methylation, Cell lineage, Embryology

## Abstract

Heterogeneous nuclear ribonucleoprotein A2/B1 (hnRNPA2/B1) plays an important role in RNA processing via in m^6^A modification of pre-mRNA or pre-miRNA. However, the functional role of and relationship between m^6^A and hnRNPA2/B1 in early embryonic development are unclear. Here, we found that hnRNPA2/B1 is crucial for early embryonic development by virtue of regulating specific gene transcripts. HnRNPA2/B1 was localized to the nucleus and cytoplasm during subsequent embryonic development, starting at fertilization. Knockdown of hnRNPA2/B1 delayed embryonic development after the 4-cell stage and blocked further development. RNA-Seq analysis revealed changes in the global expression patterns of genes involved in transcription, translation, cell cycle, embryonic stem cell differentiation, and RNA methylation in hnRNPA2/B1 KD blastocysts. The levels of the inner cell mass markers OCT4 and SOX2 were decreased in hnRNPA2/B1 KD blastocysts, whereas that of the differentiation marker GATA4 was decreased. N6-Adenosine methyltransferase METTL3 knock-down caused embryonic developmental defects similar to those in hnRNPA2/B1 KD embryos. Moreover, METTL3 KD blastocysts showed increased mis-localization of hnRNPA2/B1 and decreased m^6^A RNA methylation. Taken together, our results suggest that hnRNPA2/B1 is essential for early embryogenesis through the regulation of transcription-related factors and determination of cell fate transition. Moreover, hnRNPA2/B1 is regulated by METTL3-dependent m^6^A RNA methylation.

## Introduction

During the initial few rounds of mitotic cell division, gene expression in mammalian zygote is repressed, and the embryos rely on maternally accumulated mRNAs for their initial development. In mammals, zygotic gene transcription starts at a specific stage in embryos, such as the 2-cell stage in mouse, 4-cell stage in porcine, and 8-cell stage in human and bovine^1–4^, and it is generally described as zygotic genome activation (ZGA). After ZGA, determination of initial cell fate toward either trophectoderm (TE) or inner cell mass (ICM) begins at the morula stage of embryos, resulting in the formation of blastocysts.

In zygotic gene transcription and initial cell fate determinations, dynamic changes in epigenetic states occur. For example, initially repressed genomes from each gamete need to be reprogrammed to activate transcription in ZGA. During cell fate determination, global gene transcription must be modulated. Several epigenetic changes related to transcriptional reprogramming, including DNA demethylation and histone modification involved in ZGA and pluripotency have been extensively studied^5–8^. Although epigenetic changes and transcription level controls have been extensively studied, post-transcriptional controls in mammalian development are less clear.

mRNA in mammalian embryos undergo several post-transcriptional modifications during early embryogenesis. For example, the length of the polyadenylated tail (poly(A) tail) of mRNA in many embryos is dynamically changed during oocyte maturation and early embryogenesis, controlling the translational efficiency of mRNAs. Recently, uridylation at the 3′ end of mRNAs has been found to play a role as a signal for mRNA degradation^9^. In addition to polyadenylation and uridylation, other types of mRNA modifications have also been identified.

m^6^A modification is involved in post-transcriptional control and is widely found in various eukaryotic mRNAs^10–12^, but its functional roles remain unclear. With recent advances in technology such as methylated RNA immunoprecipitation followed by sequencing and m^6^A-seq, the biomedical significance of m^6^A RNA modification has been highlighted^13,14^. Methylation at the N6 position of adenosine is catalyzed by the methyltransferase complex, which consists of METTL3 and METTL14^15,16^, and the methyl groups are removed by demethylases, such as FTO and ALKBH5^17,18^. Recognition of m^6^A-modified effector proteins is carried out by ‘reader’ proteins, including YTHDF family proteins^19^. Recently, heterogeneous nuclear ribonucleoprotein A2/B1 (hnRNPA2/B1) has been found to mediate nuclear miRNA biogenesis, identifying *N*^6^-methyladenosine (m^6^A)^20^.

hnRNP belongs to a family of RNA-binding proteins that contributes to various forms of transcriptional control, such as alternative splicing, poly(A) tailing, mRNA stability and export, as well as to translational control. hnRNPA/B is comprised of four protein members (hnRNP A1, hnRNPA2/B1, A3, A0) involved in RNA processing. hnRNPA2/B1 is member of the hnRNPA/B family and show similarity to hnRNP A and hnRNP B. These proteins are key factors of the hnRNP core complex in mammalian cells and are involved in mRNA trafficking and telomere maintenance^21–24^. hnRNPA2/B1 is involved in pre-messenger RNA processing and shows sequence specificity in cells^25,26^. Sumoylated hnRNPA2/B1 also bind to miRNAs and control of miRNAs expression into exosomes^27^. Additionally, hnRNPA2/B1 is well-characterized as a splicing repressor for the HIV-1 and influenza A viral protein and regulate alternative splicing of various mRNAs^28,29^. From a developmental perspective, depletion of hnRNPA2/B1 affects chicken embryo development by interrupting smooth muscle differentiation^30^.

Recently, the roles of m^6^A modification by reader proteins during embryonic development have been examined. m^6^A modification has been implicated in the control of cell fate transition in mammalian embryonic stem cells^31,32^. m^6^A RNA modification and nuclear reader protein YT421-B control alternative splicing of the sex-determination gene *Sxl* upon depletion of the m^6^A methyltransferase protein Ime4, which is homologous to METTL3 in *Drosophila*^33^. The m^6^A reader protein YTHDH2 can bind to m^6^A methylated RNA and cause developmental delays in YTHDF2 knockout zebrafish embryos^34^.

However, the underlying functions of hnRNPA2/B1 in mammalian preimplantation development and their involvement in m^6^A RNA modification have not been reported. To determine the functional roles of hnRNPA2/B1 in mouse preimplantation embryo development, we investigated the effect of hnRNPA2/B1 on embryogenesis using an RNA interference (RNAi) approach. We further determined the spatial and temporal expression patterns and biological function of hnRNPA2/B1 in preimplantation embryo development.

## Results

### Heterogeneous nuclear ribonucleoprotein A2/B1 expression in preimplantation embryo development

**Figure 1:**
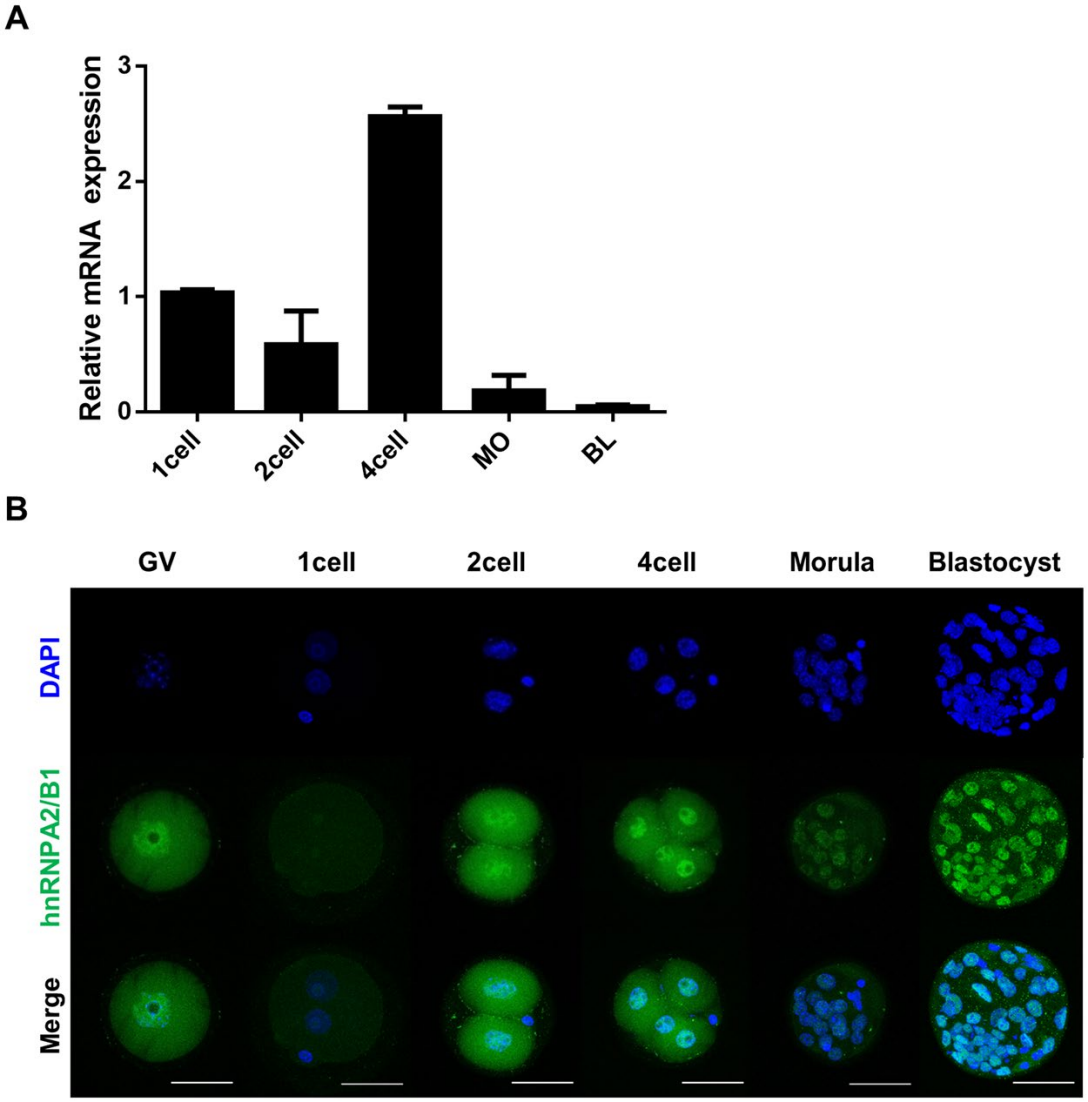
Developmental expression and localization of hnRNPA2/B1 in mouse preimplantation embryos. (**A**) Quantitative reverse-transcription polymerase chain reaction (qRT-PCR) analysis of hnRNPA2/B1 transcript levels in 1-cell (1C), 2-cell (2C), 4-cell (4C), morula (Mo), and blastocyst (BL) stages. (**C**) Immunocytochemistry (ICC) analysis revealed the hnRNPA2/B1 protein in all the nuclei during preimplantation development. Error bars indicate the mean ± s.e.m. Scale bars = 50 μm.

It has been reported that the hnRNP members localized at the nuclei of cell^35,36^, however the expression patterns and localization of hnRNPA2/B1 during early embryo development is not clear. To characterize hnRNPA2/B1 during early embryogenesis, we confirmed its gene expression patterns and localization. At the 4-cell stage, hnRNPA2/B1 gene transcription dramatically increased (Fig. 1A). The localization of hnRNPA2/B1 was detected in the nuclei and cytoplasm at all stages of the early embryo development (Fig. 1B). These results show that hnRNPA2/B1 transcription begins after ZGA and is localized in the nuclei and cytoplasm during early embryo development in mouse.

### Functional roles of hnRNPA2/B1 during mouse preimplantation development

**Figure 2:**
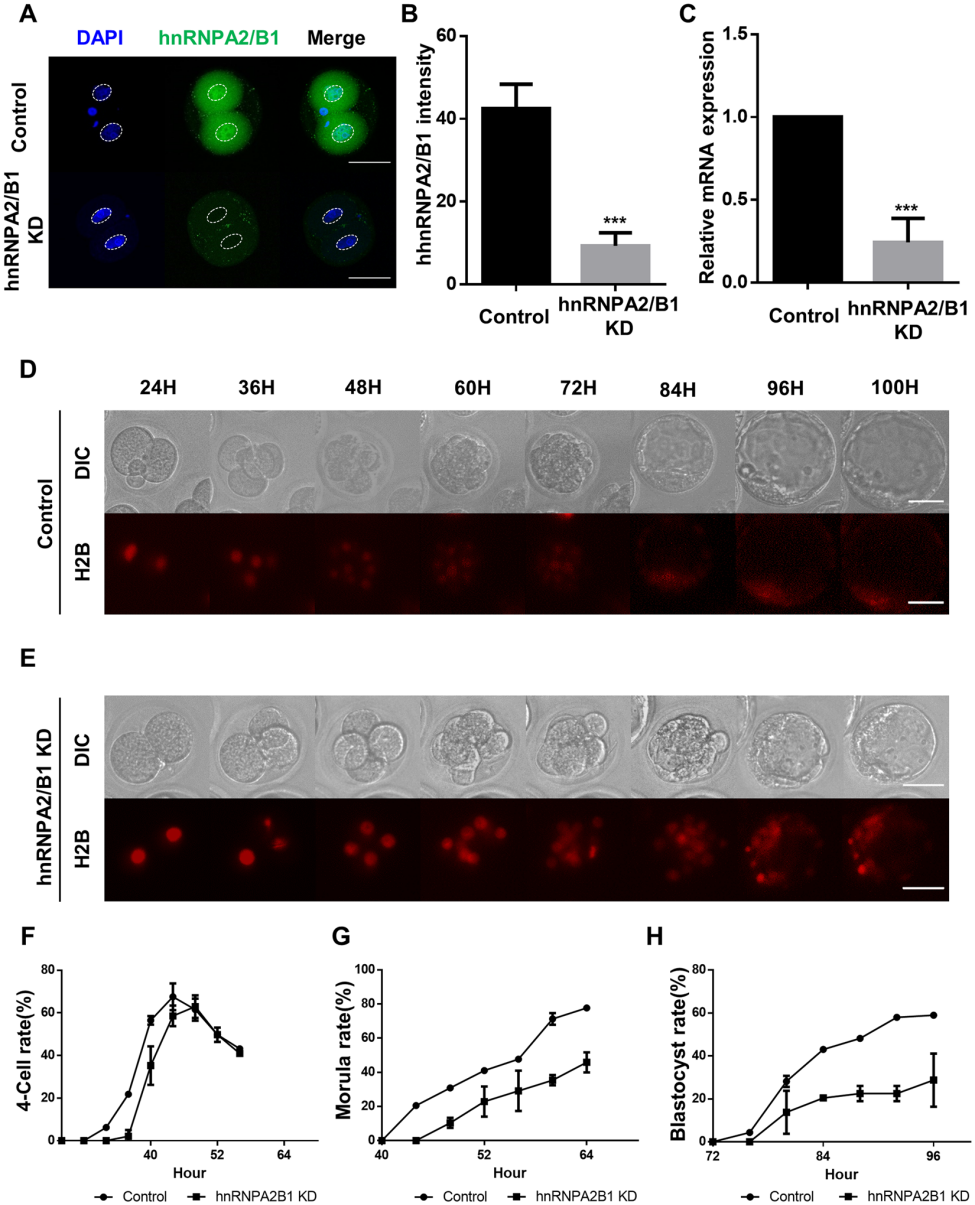
Effects of RNAi-mediated knock-down of hnRNPA2/B1 on mouse preimplantation embryo development. (**A**) Immunocytochemistry (ICC) between the control and hnRNPA2/B1 KD groups. Each embryo was microinjected at the zygote stage and cultured for 24 h. hnRNPA2/B1 intensity (**B**) and mRNA levels (**C**) were confirmed. Developmental competence between negative (**D**) and hnRNPA2/B1 KD embryos (**E**) were observed by time-lapse microscopy at 24 h after injection of eGFP dsRNA and hnRNPA2/B1 dsRNA into zygotes. DNA was visualized by cRNA encoding H2B–mCherry (red). Time-lapse experiments were repeated three times for each group. (**F**–**H**) Time course developmental changes were examined at the 4C, morula, and blastocyst stages. Developmental stages were evaluated by chromatin status. Error bars indicate the mean ± s.d. ***P < 0.01. Scale bars = 50 μm.

To investigate the cellular function of hnRNPA2/B1 during preimplantation development, we carried out knock-down of hnRNPA2/B1 transcripts during early embryo development by employing an RNAi approach. We injected 0.5 µg/µl of eGFP dsRNA or hnRNPA2/B1 dsRNA into zygotes followed by incubation for 96 h. The hnRNPA2/B1 protein was not detected in the nucleus and showed decreased signal intensity (Fig. 2A,B), and the hnRNPA2/B1 mRNA level was successfully decreased by >80% at the 2-cell stage (Fig. 2C). These results indicate that both maternal and *de novo* hnRNPA2/B1 expression was depleted. We then examined developmental competence through knock-down of hnRNAP2/B1 by time-lapse microscopy. The control embryos began to develop at the 4-cell stage after 32 h, whereas hnRNPA2/B1 KD embryos showed developmental delays until 36 h (Fig. 2D–H, Supplementary Fig. 1). hnRNPA2/B1 KD embryos also underwent development and showed retarded morula and blastocyst stages. These results indicate that the loss of hnRNPA2/B1 during embryogenesis affects the early embryo development.

**Figure 3:**
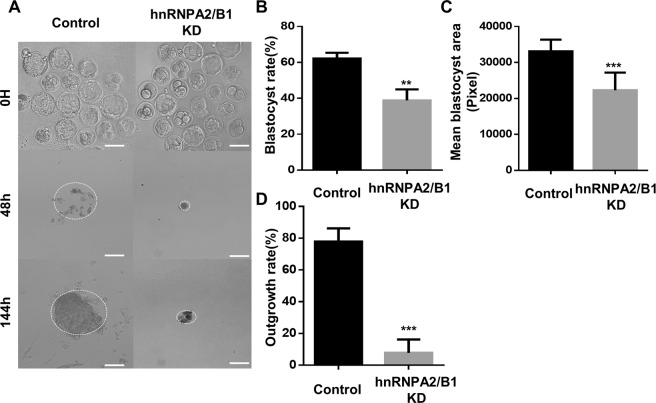
hnRNPA2/B1 is required for the development of post-implantation embryos. (**A**,**B**) Representative images showing blastocyst and outgrowth analysis using the control and hnRNPA2/B1 knockdown blastocysts. Rate of ICM-derived colony formation in the control and hnRNPA2/B1 knockdown blastocyst after 48 and 144 h. (**C**) The mean blastocyst size was calculated at 96 h. (**D**) The rate of ICM-derived colony formation in the control and hnRNPA2/B1 knockdown blastocyst after seeding at 48 and 144 h. 30–40 embryos were used in each group. The dashed line represents the area of attached colonies. Error bars indicate the mean ± s.d. **P < 0.05, ***P < 0.01. Scale bars = 100 μm.

At the blastocyst stage, the hnRNPA2/B1 groups showed decreased blastocyst rates compared with those of the control groups (Fig. 3A,B). Additionally, the mean size of hnRNPA2/B1 KD blastocysts was significantly decreased compared with that of the control blastocysts (Fig. 3C). Furthermore, the outgrowth analysis showed that hnRNPA2/B1 KD blastocysts did not form colonies (Fig. 3D). These results indicate that hnRNPA2/B1 affects blastocyst quality and post-implantation development.

### hnRNPA2/B1 affects ICM formation by regulating pluripotency in blastocysts

**Figure 4:**
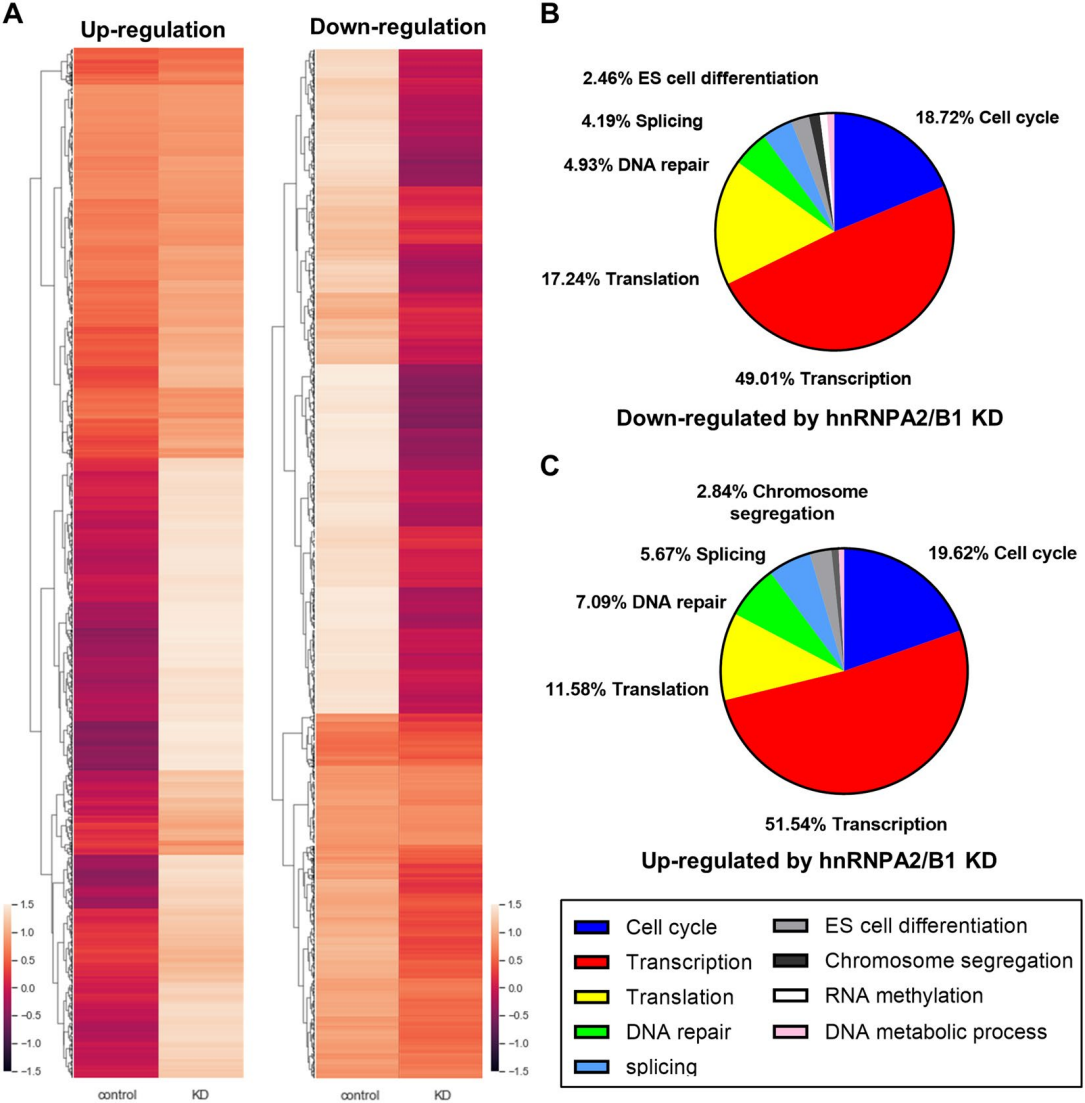
RNA-Seq analysis of the control and hnRNPA2/B1 KD blastocysts. (**A**) Heatmap comparing the transcription level of up-regulated genes or down-regulated genes at the blastocyst stage. The expression level of genes was significantly (FRKM > 2, or <2) increased or decreased compared with that of the control. Con: Control, KD: hnRNPA2/B1 KD. (**B**,**C**) Composition of up-regulated and down-regulated genes in hnRNAP2/B1 KD blastocyst at 96 h. A total of 5,846 genes was downregulated upon hnRNPA2/B1 KD, and over 45% of which are transcription-related genes. A total of 6,060 genes was up-regulated upon hnRNPA2/B1 KD, and over 50% of which are transcription-related genes.

Previous studies have showed that hnRNPA2/B1 affects key transcription factors related to pluripotency in human ES cells^37^ and interacts with Sox2 in bovine embryos based on RNA sequencing analysis^38^. Thus, we confirmed the expression of pluripotency-related proteins in hnRNPA2/B1 KD blastocysts. Consistent with the RNA-Seq results, the cells positive for the pluripotency marker proteins OCT4 and SOX2 in ICM were decreased in hnRNPA2/B1 KD blastocysts (Fig. 4A–D). Furthermore, a member of the GATA family of zinc-finger transcription factors, GATA4, was decreased in the primitive endoderm (PrE) (Fig. 4E,F). These results show that hnRNPA2/B1 regulates pluripotency-related gene expression and early cell fate determination.

### Transcriptome analysis by RNA-seq at the blastocyst stage

**Figure 5:**
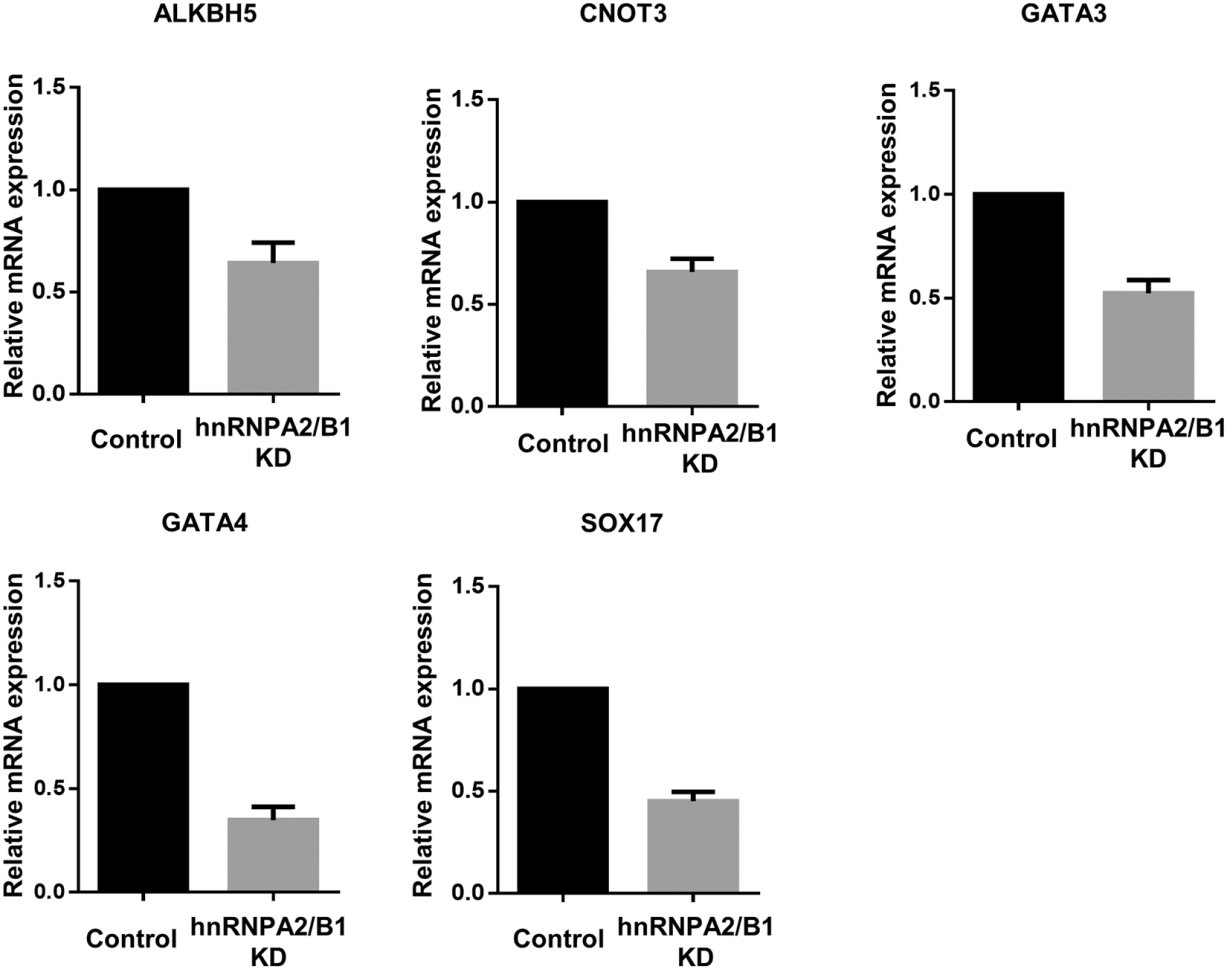
Gene expression determined by quantitative real-time PCR analysis of various gene mRNAs in the control and hnRNPA2/B1 KD embryos at the blastocyst stage. Three experimental replicates were carried out. ***P < 0.01. Error bars indicate the mean ± s.e.m.

**Figure 6:**
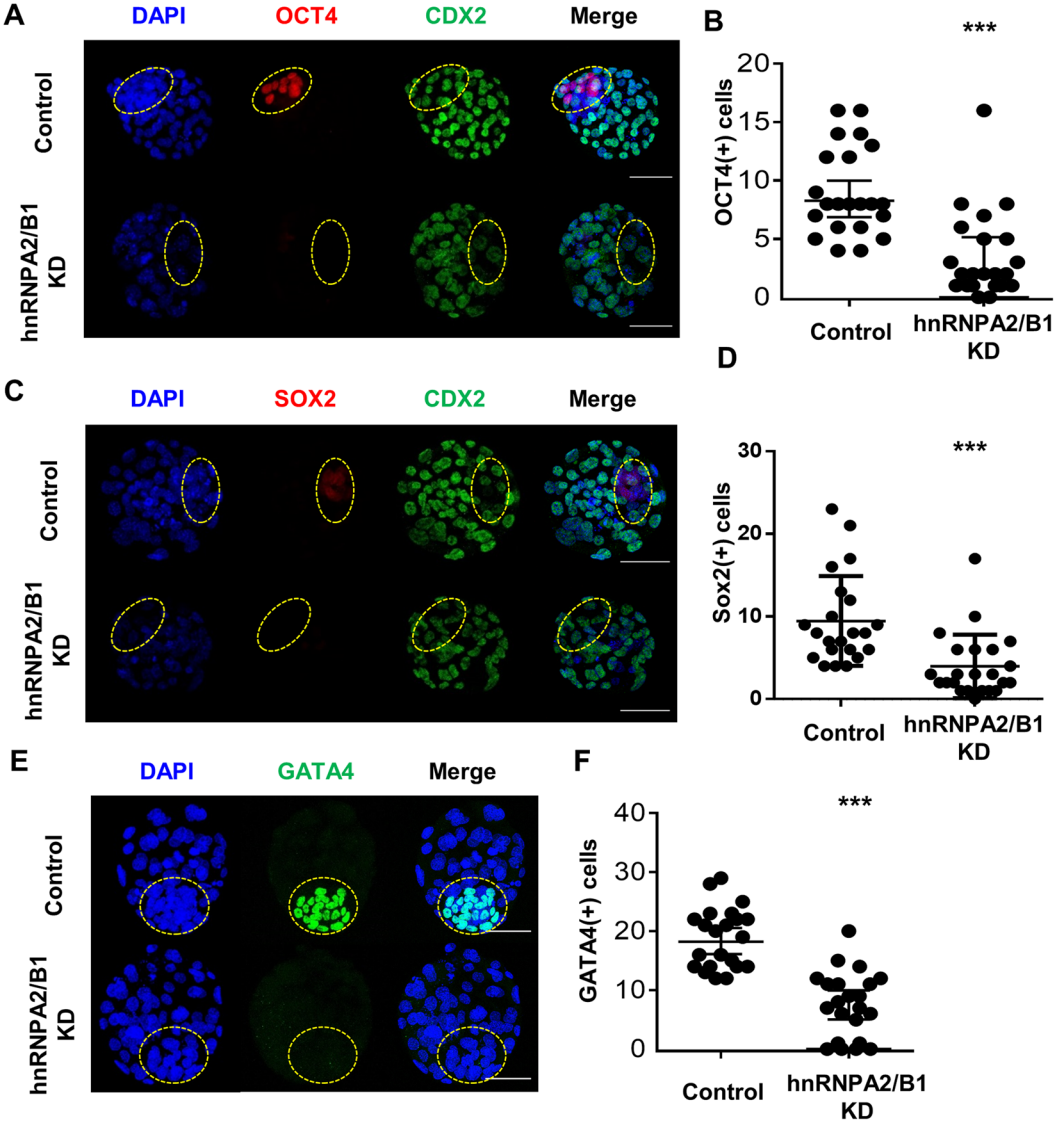
Effects of hnRNPA2/B1 knockdown on pluripotency-related gene expression. **(A**,**B**) OCT4 and CDX2 and (**C**,**D**) SOX2 and CDX2 double-immunostaining in the control and hnRNPA2/B1 KD blastocysts (red: OCT4, red: SOX2, green: CDX2, blue: DNA). 5–10 embryos were used in each experiment (n = 3). (**E**,**F**) GATA4 immunostaining in the control and hnRNPA2/B1 KD blastocyst (green; GATA, blue; DNA). The dashed line represents area of the ICM. Error bars indicate the mean ± s.e.m. ***P < 0.01. Scale bars = 50 μm.

To determine the causes of developmental delay and cell fate in hnRNPA2/B1 knock-down embryos, we performed RNA-seq analysis at the blastocyst stage. We obtained a list of approximately 12 000 genes showing ≥ 2-fold down or up-regulation and compared with the controls the gene Ontology (GO) analysis involved classification of each gene (Fig. 5A). The list of up-regulated genes included those involved in transcription, DNA repair, splicing, and RNA methylation. The down-regulated genes showed results similar to those of the up-regulated genes, with increased translation; particularly, the expression of ES cell differentiation gene group was decreased. Based on GO term analysis, genes whose expression were up- or down-regulated by hnRNPA2/B1 KD were selected (Fig. 5B,C). The RT-PCR results showed that m6A demethylase ALKBH5, CCR4-NOT complex 3, and epiblast-related genes (GATA3, GATA4 and SOX17) were down-regulated at the blastocyst stage (Fig. 6). These results demonstrate that hnRNPA2/B1 regulates global gene expression patterns in embryo development.

### METTL3 is required for m^6^A RNA methylation and hnRNPA2/B1 localization during mouse preimplantation development

**Figure 7:**
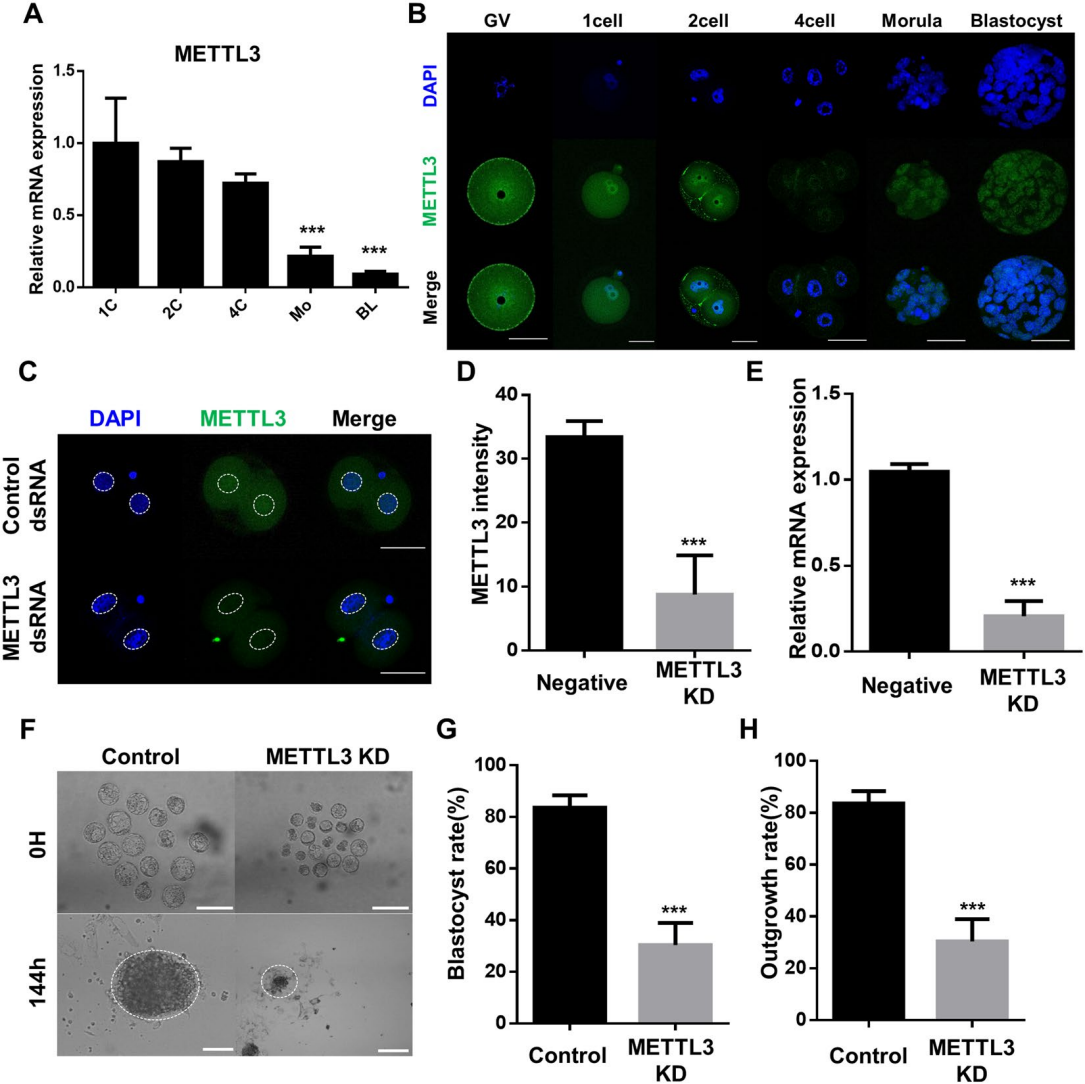
Effect of METTL3 during the early embryo development. (**A**) mRNA expression levels analysed by qRT-PCR and (**B**) localization by ICC GV at the blastocyst stage. (**C**) ICC of METTL3 in the control and METTL3 KD 2-cell embryos. Each embryo was microinjected at the zygote stage and cultured for 24 h. (**D**) METTL3 intensity and (**E**) mRNA levels were examined to evaluate the efficiency of METTL3 KD. (**F**) Representative images showing blastocyst and outgrowth analysis using the control and METTL3 knockdown blastocysts. (**G**) Rate of blastocyst and (**H**) ICM-derived colony formation in control and METTL3 knockdown blastocysts after 144 h. Three experimental replicates were examined using 20–30 embryos per group. The dashed line represents the area of nuclei. ***P < 0.01. Error bars indicate the mean ± s.d.

**Figure 8:**
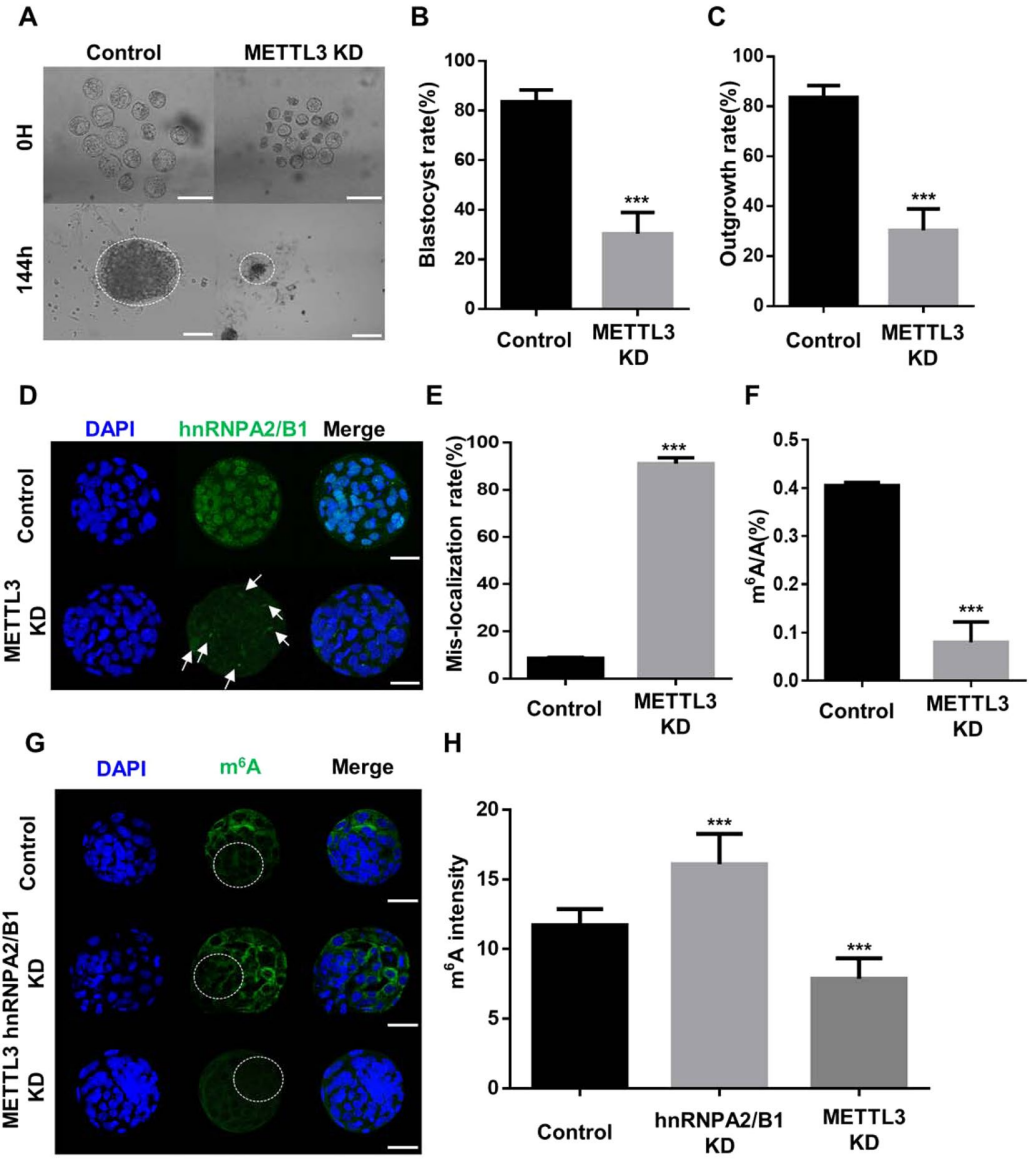
Effects of RNAi-mediated knockdown of METTL3 on m^6^A RNA methylation and hnRNPA2/B1 localization. (**A**) ICC analysis of N6-methyladenosin (m^6^A) in the control and METTL3 KD 4-cell embryos. METTL3 localized in the cytoplasm and decreased m^6^A intensity by METTL3 KD embryos. (**C**,**D**) ICC of m^6^A images in the control, hnRNPA2/B1 KD, and METTL3 KD 4D blastocysts. (**E**,**F**) Immunocytochemistry detection of hnRNPA2/B1 in the control and METTL3 KD blastocysts. 30–40 embryos were used in each group. ***P < 0.01. Error bars indicate the mean ± s.d. Scale bars = 50 μm.

Previous studies have showed that hnRNPA2/B1 bind to m^6^A and the METTL3-14 complex has a catalytic effect following methylation of the N-6 position of adenosine in mammals^16,20^. In the METTL3-14 complex, METTL3 mainly exerts is function through the m^6^A catalytic core^39^, but the function of METTL3 and relationship between m^6^A hnRNPA2/B1 and METTL3 in early embryo development are unknown. Thus, we investigated the roles of METTL3 using an RNAi approach. The transcript level of METTL3 was decreased and its protein was localized in the nucleus and cytoplasm during the early embryo development (Fig. 7A,B, Supplementary Fig. 2). METTL3 KD embryos presented decreased levels of mRNA and proteins (Fig. 7C–E). Blastocyst was not formed and there was no outgrowth in METTL3 KD embryos (Fig. 7F–H). These results indicated that KD of METTL3 causes developmental defects. To confirm the relationship between m^6^A and METTL3, ICC was conducted to confirm the localization and levels of m^6^A by KD of an m^6^A regulator. In 4-cell stage, m^6^A was distributed in the cytoplasm and not in the nucleus (Fig. 8A). KD of METTL3 embryos decreased METTL3 signal intensity (Fig. 8B). Particularly, m^6^A was detected in the trophectoderm, and not in the ICM region, at the 4D blastocyst stage (Fig. 8C). METTL3 KD blastocysts also showed a decreased intensity of m^6^A but increased intensity in hnRNPA2/B1 KD blastocysts (Fig. 8D). In METTL3 KD embryos, m^6^A transcript quantification level using isolated total RNA was significantly decreased compared with that in the controls. (Supplementary Fig. 3). In KD of METTL3 blastocysts, hnRNPA2/B1 intensity was decreased in the nucleus and accumulated in the cytoplasm, which differed from control blastocysts (Fig. 8F,G). These results suggest that the depletion of METTL3 induces developmental defects and causes reducing the levels of hnRNPA2/B1 and m^6^A transcripts.

## Discussion

The ZGA process is important during early embryonic development; particularly, RNA synthesis is involved in differentiation into ICM/TE and formation of the blastocyst. hnRNPA2/B1 is an RNA-binding protein and a member of the hnRNP family, and contains several RNA recognition motif (RRM) sites that can bind to mRNAs and regulate various mRNA processes such as mRNA transport, alternative splicing, and maintenance of mRNA stability^22,23,40^. In embryonic stem cells, hnRNPA2/B1 is crucial for stabilizing pluripotency by regulating key pluripotency-related proteins; however, the functional roles of hnRNPA2/B1 during mammalian early embryogenesis have not been widely examined. In this study, we demonstrated that hnRNPA2/B1 affects early embryo development by regulating mRNA transcription. Furthermore, hnRNPA2/B1 is required for the ICM/TE transition during early embryo development and relationship between hnRNPA2/B1 and m^6^A methylation by METTL3.

In the present study, we found that the hnRNPA2/B1 transcript is highly upregulated during the 2-cell to 4-cell stages at a time close to that when ZGA occurs. Furthermore, early embryo developmental delay was observed after the 4-cell stage in KD of hnRNPA2/B1 and decreased blastocyst rates and quality were detected. Previous studies have revealed that the depletion of RNA-binding proteins causes embryo developmental defects. Eif2c2 (one argonaute family member)-deficient mice exhibited developmental arrest in early embryonic stages^41^. Depletion of the THO complex resulted in the failure of blastocyst development via inhibition of pluripotency-related gene transcription^42^. Thoc1/Hpr1/p84 depleted embryos also showed interrupted blastocyst development^43^. Similar to these RNA-binding protein developmental defects, hnRNPA2/B1 phenotypes during early embryo development were accompanied by regulation of gene expression. These transcripts variations may affect mRNA processing after ZGA. The RNA-Seq data revealed that the genes up-/down-regulated by knock-down of hnRNPA2/B1 were related to transcription, translation, cell cycle, splicing, and RNA methylation. These results indicate that hnRNPA2/B1 has important roles in regulating mRNA transcription and translation during early embryo development by affecting specific transcription factors. YTHDC1, an m^6^A reader, localizes in the nucleus during oocyte to blastocyst development and has crucial roles in mouse oocyte maturation and embryo viability by regulating alternative polyadenylation and splicing^31^. In zebrafish embryo, depletion of the m^6^A reader protein YTHDF2 caused developmental delays by affecting pre-mRNA synthesis in the maternal to zygotic transition^34^. During germ cell meiosis, YTHDC2 modulates the levels of m^6^A-enriched transcripts to maintain the gene expression program^44^. The results of the present study elucidated the roles of hnRNPA2/B1 in early embryo development and genes affected by hnRNPA2/B1 expression; however, the exact mechanism related to post-transcriptional control by hnRNPA2/B1remains unclear. Further studies are necessary to determine the contribution of hnRNPA2/B1 and m^6^A-mediated gene transcription during ZGA and further cell fate determination during embryogenesis.

In our results, developmental defect was observed and blastocyst formation failed following KD of METTL3 (Fig. 7F–H). Recent studies have showed that m^6^A and methyltransferase complex (METTL3-14complex) have crucial roles in mammalian spermatogenesis^45,46^. Furthermore, METTL3-deficient mice exhibited early embryonic lethality with defects in the regulation of cell fate determination^32^. In embryonic stem cells, m^6^A and its methyltransferase MEETL3 regulate self-renewal by maintaining the mRNA stability of pluripotency-related genes^19^. METTL3 KD blastocysts also showed increased mis-localization of hnRNPA2/B1 (Fig. 6D,E), with reduced levels of m^6^A RNA methylation after the 4-cell stage (Fig. 8A–D). These results suggest that the localization of hnRNPA2/B1 is affected by METTL3 and crucial for embryo development.

Our results showed that the development of hnRNPA2/B1 KD embryos was delayed and that the developed blastocysts could not form colonies under embryonic stem cell conditions (Fig. 2). These blastocysts were morphologically altered, and few ICM cells were positive for OCT4, SOX2, and GATA4 (Fig. 4). The RNA-Seq data showed that several transcription factors were up- or down-regulated in hnRNPA2/B1 KD blastocysts (Fig. 5). Among the down-regulated genes, signal transducers and activators of transcription showed a similar phenotype^19^. In mouse ESCs, hnRNPA2/B1 KD blastocysts could not maintain pluripotency and self-renewal because of decreased pluripotency and cell cycle-related gene expression^37^. The binding of m^6^A reader protein to m^6^A promotes de-adenylation and mRNA decay by directly recruiting the CCR4-NOT complex, a de-adenylase complex^47^. We confirmed that CNOT3 is down-regulated in hnRNPA2/B1 KD embryos (Fig. 6). Depletion of CNOT3, a component of the Ccr4-nordestenylase complex, also affected the number of OCT4-positive cells and impaired epiblast maintenance^48^. The m^6^A-Seq data for mESC revealed that the m^6^A targets include the pluripotency network and transcripts with dynamically controlled abundance; m^6^A is related to cell fate determination^19^. m^6^A RNA immunoprecipitation sequencing (RIP-Seq) results for mESC revealed the core pluripotency regulators including Nanog, klf4, Myc, and Lin28^49^. The phenotype following downregulation of these genes cannot be attributed to hnRNPA2/B1 and m^6^A modification directly, this may involve multiple effects of the reduced levels of genes involved in regulation of RNA processing.

In summary, our results demonstrate that hnRNPA2/B1 is required for early embryonic development, particularly for pluripotency-related gene expression. Moreover, METTL3 is also essential for early embryo development via regulation of m^6^A transcripts.

## Materials and Methods

### Reagents

All chemicals were purchased from Sigma-Aldrich (St. Louis, MO, USA) unless stated otherwise.

### Mice

Female 6–8 weeks-old and Male 8–12 weeks old CD1(ICR) mouse strains were purchased from Orientbio (Gapyeong, Kyeonggi, Korea). All animal manipulations were approved and conducted according to the guidelines of the Animal Research Committee of Chungbuk National University (CBNUA-1026-16-01) and conducted according to the Standard Operation Procedures (SOP) of the Laboratory Animal Research Center, CBNU.

### Mouse embryo collection and culture

To retrieve the zygotes, 6–8 weeks female superovulated ICR mice were injected with 5 U pregnant mare’s serum, followed by injection of 5 U of human chorionic gonadotropin (hCG) at 46–48 h. The female mice were then sacrificed at 16–18 h post-hCG injection. Zygotes with cumulus cells were denuded by incubation with 200 μL/mL hyaluronidase for 1 min. The zygotes were cultured in 20-μL drops of KSOM media, covered with mineral oil, and incubated at 37 °C and 5% CO_2_. The collection of zygotes for *in vitro* experiments was based on scoring of the formation of visible pronuclei at 20 h post-hCG injection. Early embryo stage sampling times were as follows: 1-cell stage, 23 h post-hCG; 2 cell stage, 40 h; 4-cell stage, 52 h; Mo, 80 h; and BL, 96 h. Bright field images of embryos were taken using a Nikon TE 2000 inverted microscope at ×100 (Tokyo, Japan). Mean blastocyst diameters were calculated from these data and measured using ImageJ by pixel.

### Immunocytochemistry and confocal microscopy

Mouse oocytes and embryos were washed with DPBS containing 1 mg/mL polyvinyl alcohol and fixed for 1 h in 3.7% (w/v) paraformaldehyde in DPBS. The fixed embryos were then permeabilized with 0.2% (v/v) Triton X-100 in DPBS for 1 h at 20 °C. The embryos were incubated with the primary antibody (hnRNPA2/B1: a mouse polyclonal antibody, Abcam, Cambridge, UK; ab 6102, METTL3: a rabbit polyclonal, Abcam; ab 195352, OCT4: a rabbit polyclonal, Santa Cruz Biotechnology, Dallas, TX, USA; sc-8628, Sox2: a rabbit polyclonal, Santa Cruz; sc-17320, GATA4: a rabbit polyclonal, Abcam; ab 84593, m^6^A: a rabbit polyclonal, Synaptic Systems, Goettingen, Germany, 202–003) antibody overnight, followed by a fluorescein isothiocyanate- or Rhod-labeled secondary antibody (Santa Cruz). Finally, the nuclei were stained with Hoechst 33342 and mounted on a glass slide with Vectashield. Between each step, the embryos were washed with DPBS containing 1 mg/mL polyvinyl alcohol three times for 10 min each. All oocytes and embryos were examined using a confocal laser-scanning microscope (Zeiss LSM 710 META, Jena, Germany) with a 40 × water-immersion objective lens. Quantification of 2-cell embryos for knock-down efficiency was carried out in the nucleus of each blastomere. Cytoplasmic regions were selected without the nucleus to quantify m^6^A intensity in the blastocysts.

### Quantitative real-time PCR

Embryos were washed with phosphate-buffered saline (PBS), snap-frozen in liquid nitrogen, and stored at −80 °C. mRNA was extracted using the Dynabeads mRNA Direct Kit (Invitrogen, Carlsbad, CA, USA) according to the manufacturer’s instructions. cDNA was synthesized by reverse transcription using the LeGene Express 1 Strand cDNA Synthesis master mix (LeGene, San Diego, CA, USA). The mRNA expression of several genes was detected by quantitative real-time PCR (qRT-PCR) with specific primer pairs (Table 1). The PCR was performed according to the instructions of the real-time PCR machine manufacturer Bio-Rad CFX connect (Bio-Rad Laboratories, Hercules, CA, USA). Reactions were conducted according to the protocol provided with the RNA using Wiz pure qPCR super green master mix (Wiz Pure, Grand Island, NY, USA). The PCR was performed as follows: denaturation at 95 °C for 10 min, 40 cycles of amplification and quantification at 95 °C for 10 s, 55 °C or 60 °C for 30 s, and 72 °C for 30 s with a single fluorescence measurement, melting at 65 °C–95 °C with a heating rate of 0.2 °C/s, and continuous fluorescence measurement and cooling to 12 °C. GAPDH was used as the internal control in all the experiments.

**Table 1:** Primer sequences for qRT-PCR.

Gene	GenBank accession No.	Sequence (5′– 3′)	Amplicon size (bp)
hnRNPA2/B1 dsRNA	NM-016806.	F: TAATACGACTCACTATAGGGAGACCACTGAGCCAAAACGTGCTGTAG R: TAATACGACTCACTATAGGGAGACCACCAGGACCATAGTTCCCTCCA	623
METTL3 dsRNA	NM-019721.2	F: TAATACGACTCACTATAGGGAGACCACTGAGCCAAAACGTGCTGTAG R: TAATACGACTCACTATAGGGAGACCACCAGGACCATAGTTCCCTCCA	628
HnRNPA2/B1	NM-016806.	F: CCGATAGGCAGTCTGGAAAG R: TATAGCCATCCCCAAATCCA	300
METTL3	NM-019721.2	F: TAGCATCTGGTCTGGCCTCT R: TCACTGGCTTTCATGCACTC	286
ALKBH5	NM-172943.4	F: CTCAGTGGGTATGCTGCTGA R: CCGGTTTTCTTCTTTGTCCA	279
CNOT3	XM-006539757.3	F: GGCAAAAGCTCCACAATGCA R: CTTCAAGCCCTCAATCCGGT	428
GATA3	NM-008091.3	F: GCTACGGTGCAGAGGTATCC R: GCGGATAGGTGGTAATGGGG	463
GATA4	NM-001310610.1	F: CCCTGGAAGACACCCCAATC R: TTTGAATCCCCTCCTTCCGC	359
SOX17	NM-001289464.1	F: TAGGCAAGTCTTGGAAGGCG R: GCATAGTCCGAGACTGGAGC	475

(F: forward. R: reverse).

### Double-stranded RNA injection time-lapse microscopy

Knock-down of hnRNPA2/B1 and METTL3 in mouse embryos was performed via microinjection of double-stranded RNA (dsRNA) into the zygotes. hnRNPA2/B1 and METTL3 dsRNAs were designed using information obtained from the National Center for Biotechnology Informationdatabase (hnRNPA2/B1: NM-016806.3, METTL3:NM_019721.2) as previously described^50^. The dsRNA was resuspended in nuclease-free water, and microinjection was performed using an injection pipette and inverted microscope (Nikon TE2000U, Tokyo, Japan) equipped with a micromanipulator (Eppendorf, Hamburg, Germany). To microinject a constant amount of dsRNA, an injection pipette was connected to a Femto Jet (Eppendorf). The embryos were transferred to 10-μL drops of M2 medium. The embryos were held in place with a holding pipette, and the plasma membrane was penetrated by the injection pipette with constant dsRNA diluted in nuclease-free water. To assess injection damage, the zygotes were infected with eGFP dsRNA as the positive control. Injected embryos were cultured in KSOM at 37 °C with 5% CO_2_. To confirm the delay in the developmental rate of hnRNPA2/B1 KD embryos, eGFP dsRNA and H2B-mcherry mRNA were mixed, and the microinjected zygotes were placed on the Lumascope 620 (Etaluma Inc. Carlsbad, CA) inverted microscope installed inside an incubator maintained at 37 °C and 5% CO_2_. Images were captured at 5-min intervals for 100 h.

### Blastocyst outgrowth analysis using blastocyst

For outgrowth analysis, we removed the zona pellucida of 96 h *in vitro* cultured blastocysts using acid Tyrode’s solution. The culture medium was used DMEM supplemented with 2 mM L-glutamine, 100 μM MEM non-essential amino acid solution, β-mercaptoethanol, 1% penicillin and streptomycin, 10% fetal bovine serum, and 1000 U/mL murine leukemia inhibitory factor. Nicked blastocysts were transferred to 96-well culture dishes coated with gelatin for outgrowth analysis. We examined the outgrowth rate at 48 and 144 h.

### Isolation of RNA from blastocyst for RNA-Seq analysis and GO term analysis of differentially expressed genes

Control and hnRNPA2/B1 KD blastocysts (day 4) were washed with PBS-BSA three times. Seventy blastocysts were transferred to each e-tube and the supernatant was removed. Each tube was stored at −80 °C before isolation of total RNA. Isolation and purification of total RNA from each sample were carried out using the NucleoSpin RNA XS Kit (Clontech, Mountain View, CA, USA). For each time point, 70 of day 4 blastocysts were collected to ensure the yield of RNA and that the samples were representative. Sequencing was carried out on an Illumina HiSeq 2500 according to the manufacturer’s instructions (San Diego, CA, USA). After obtaining library data, the sequenced reads were mapped and quantified using Kallisto software. Mouse genome reference used was mm10 from USCS. Differential gene expression values were sorted by ± 2-fold changes, and then filtered by up-regulation (6060 genes) and down-regulation (5846 genes). Heat map was generated using Pandas software (https://pandas.pydata.org/). Ensemble gene IDs were translated into official gene symbol IDs with BioMart. Differential gene expression values were then filtered by ± 2-fold change and heatmap of gene expression was generated using Pandas software (https://pandas.pydata.org/). The GO term annotation data were downloaded from Gene Ontology Consortium (http://geneontology.org/) and were processed to retrieve the data of all transcripts and its corresponding GO term association.

### Quantification of m^6^A RNA methylation

To quantify m^6^A methylated RNA, we used the m^6^A RNA Quantification Kit (EpiGentek, Farmingdale, NY, UA). Total RNA was isolated from 100 blastocysts using TRIzol and quantified using Nanodrop (Thermo Fisher scientific, Wilmington, DE, USA). Briefly, 2 μL of negative control (NC), 2 μL of positive control (PC), and 200 ng of sample RNA were added to 80 μL of binding solution in each strip well, and then the plate was incubated at 37 °C for 90 min. For m^6^A RNA capture, 1:1000 dilution capture antibody was added to each well and incubated at room temperature (RT) for 60 min followed by incubation with 1:2000 detection antibody at RT for 60 min, and with 1:5000 enhancer buffer at RT for 30 min. After the addition of detection solution (100 μL) to each well, the plate was incubated at RT for 10 min in the dark. After adding stop solution (100 μL), each well was read on a microplate Sunrise reader (Tecan, Männedorf, Switzerland) at 450 nm. Between each step, the plate was washed several times with 1X wash buffer.

## Supplementary information

Supplementary figure 1, Supplementary figure 2, Supplementary figure 3
