# Supplementary material for: Functional roles of hnRNPA2/B1 regulated by METTL3 in mammalian embryonic development
Source: Sci Rep. 2019 Jun 14;9:8640. doi: 10.1038/s41598-019-44714-1 (PMC6572863; doi:10.1038/s41598-019-44714-1)
Supplement: Supplementary file 1 — Supplementary figure 1, Supplementary figure 2, Supplementary figure 3 [file 41598_2019_44714_MOESM1_ESM.pdf]

Supplementary Information's for

Functional roles of hnRNPA2/B1 regulated by METTL3 in mammalian embryonic development

Jeongwoo Kwon[1], Yu-Jin Jo[2], Suk Namgoong[1 *] and Nam-Hyung Kim [1 *]

[1]Department of Animal Sciences, Chungbuk National University, Gaesin-dong, Cheongju, Chungbuk. 361-763, Republic of Korea

[2]Primate Resources Center (PRC), Korea Research Institute of Bioscience and Biotechnology (KRIBB), Jeongeup-si, Jeollabuk-do, 56216, Republic of Korea

[*]Correspondence to Suknamgoong@gmail.com or nhkim@chungbuk.ac.kr

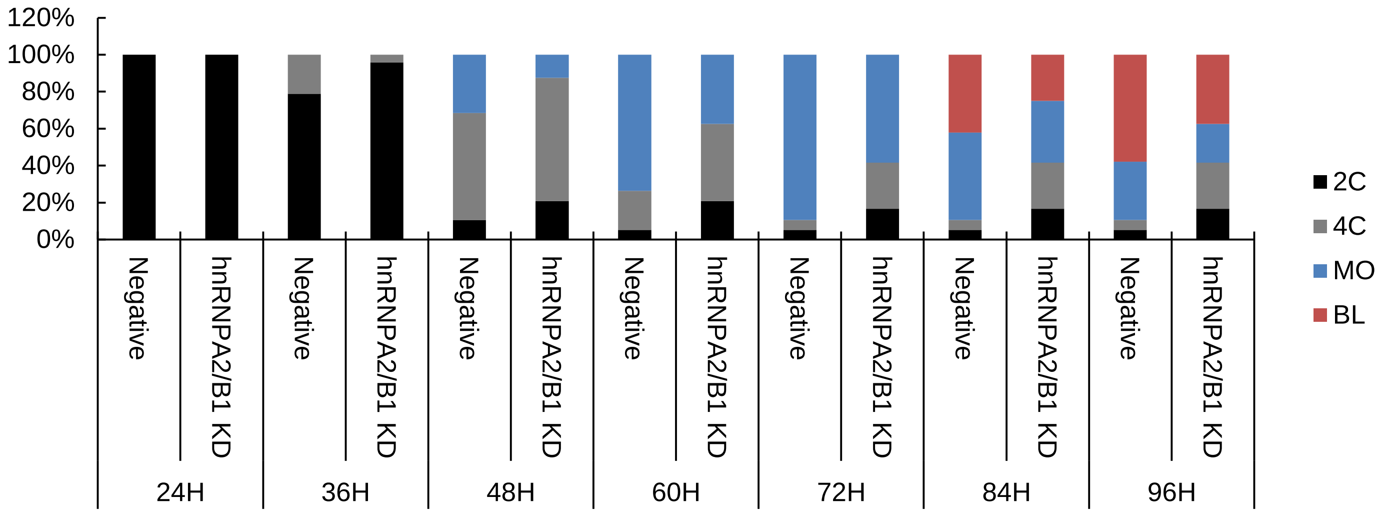

Supplementary Figure 1. Developmental delay of hnRNPA2/B1 KD embryos. Developmental competence was observed by time-lapse microscopy at 24 h after injection of eGFP dsRNA (negative) and hnRNPA2/B1 dsRNA (hnRNPA2/B1 KD) into zygotes. Experiments were repeated three times for each group. Developmental stage was evaluated at the 2C, 4C, morula, and blastocyst stages and evaluated by chromatin status.

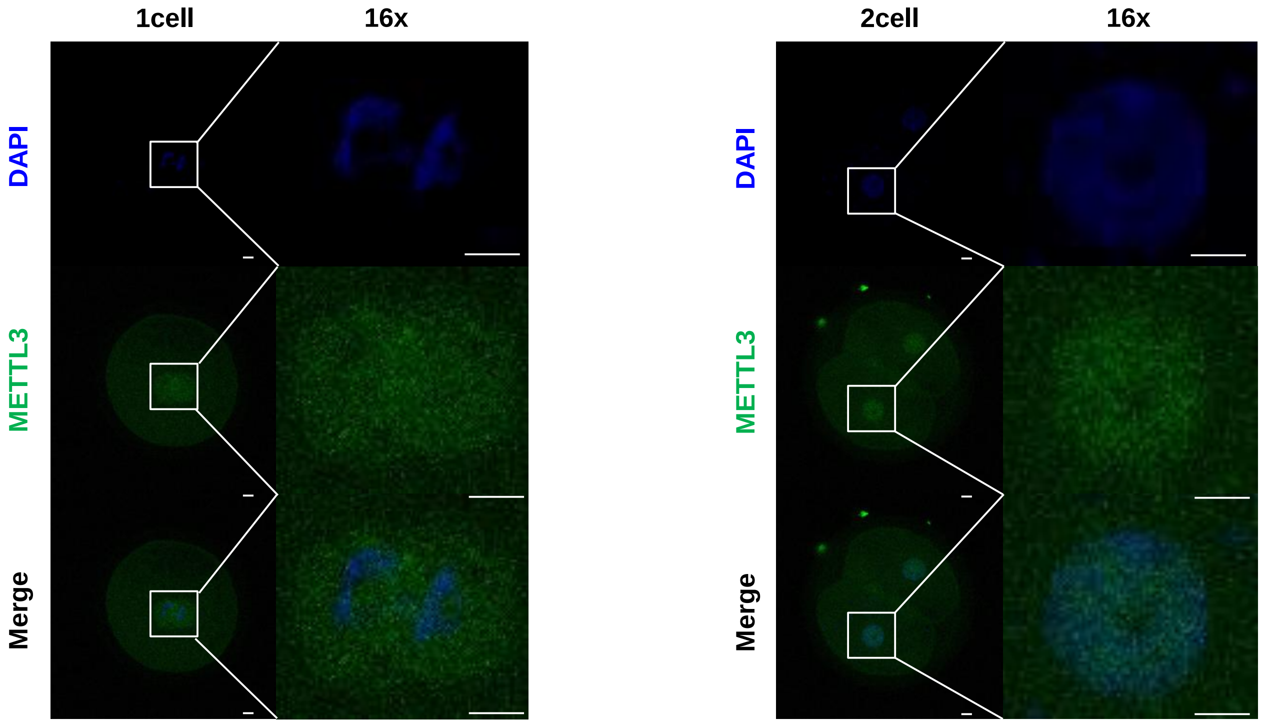

Supplementary Figure 2. Localization of METTL3 in 1cell and 2cell embryos using confocal microscopy. METTL3 localized at the nucleus during early embryo development. Scale bar = 10 μm

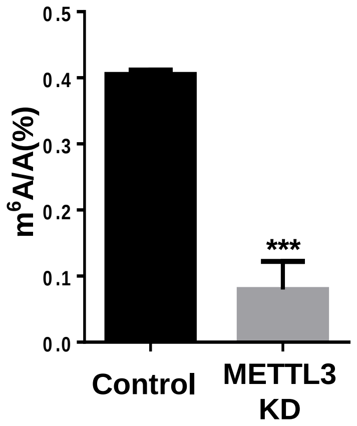

Supplementary Figure 3. Quantification of m6A RNA methylation in control and METTL3 knockdown blastocysts. One hundred blastocysts were examined in each group for total RNA isolation.
